# Very long‐chain acyl‐CoA dehydrogenase deficiency in a Swedish cohort: Clinical symptoms, newborn screening, enzyme activity, and genetics

**DOI:** 10.1002/jmd2.12268

**Published:** 2022-01-09

**Authors:** David Olsson, Michela Barbaro, Charlotte Haglind, Maria Halldin, Svetlana Lajic, Sara Tucci, Rolf H. Zetterström, Anna Nordenström

**Affiliations:** ^1^ Department of Women's and Children's Health, Unit for Pediatric Endocrinology and Metabolic Disorders Karolinska Institutet/Karolinska University Hospital Stockholm Sweden; ^2^ Center for Inherited Metabolic Diseases, CMMS Karolinska University Hospital Stockholm Sweden; ^3^ Department of Molecular Medicine and Surgery Karolinska Institutet Stockholm Sweden; ^4^ Department of General Pediatrics, Adolescent Medicine and Neonatology Medical Centre‐University of Freiburg, Faculty of Medicine Freiburg Germany

**Keywords:** clinical outcome, newborn screening, Sweden, very long‐chain acyl‐CoA dehydrogenase deficiency (VLCADD)

## Abstract

Very long‐chain acyl‐CoA dehydrogenase deficiency (VLCADD) is a recessive disorder of fatty acid beta‐oxidation with variable phenotype. Patients may present during the neonatal period with lethal multi‐organ failure or during adulthood with a myopathic phenotype. VLCADD is included in the Swedish newborn screening (NBS) program since 2010. The study describes the phenotype and biochemical findings in relation to the genotype, enzyme activity, and screening data in a Swedish cohort of pediatric patients with VLCADD. A total of 22 patients (20 diagnosed via NBS between 2010 and 2019, two diagnosed pre NBS) were included. Parameters analyzed were enzyme activity (palmitoyl CoA oxidation rate); *ACADVL* genotype; NBS results including Collaborative Laboratory Integrated Reports (CLIR) score; biochemical findings; treatment; clinical outcome. A clinical severity score (CSS) was compiled using treatment interventions and clinical symptoms. A possible correlation between CSS and VLCAD residual enzyme activity and NBS CLIR score was analyzed. The most common *ACADVL* variant (c.848T>C) was identified in 24/44 alleles. Five novel variants were detected. Clinical manifestations varied from asymptomatic to severe. There was a correlation between CSS, residual enzyme activity, and CLIR scores. Most patients diagnosed via NBS had less severe disease compared to those clinically diagnosed. In conclusion, the identified correlation between the NBS CLIR score, residual enzyme activity, and clinical outcome suggests that information available neonatally may aid in treatment decisions.

**Synopsis**

Swedish patients with VLCADD identified via NBS show milder symptoms compared to clinically diagnosed patients.

## 1 INTRODUCTION

Very long chain acyl‐CoA dehydrogenase deficiency (VLCADD) (OMIM #201475) is an autosomal recessive disorder of fatty acid beta oxidation.^1^ The enzyme encoded by the *ACADVL* gene catalyzes the first step in the mitochondrial beta oxidation of very long chain fatty acids. Due to limited ability to use fatty acids as energy substrate, patients with VLCADD are at risk of developing metabolic decompensation in situations with increased energy demand or catabolism such as infections with fever or vomiting, or periods of prolonged fasting. In case of neonatal onset, VLCADD can result in potentially lethal metabolic decompensation with multiorgan failure. Milder phenotypes including the late onset myopathic form have a low mortality rate and symptoms occur primarily after exercise.^2^, ^3^, ^4^, ^5^

Treatment consists of prevention of catabolism, limitation of dietary intake of long chain fatty acid triglycerides (LCT), and dietary supplementation with medium chain triglycerides (MCT) and essential fatty acids (EFA). Published treatment recommendations for symptomatic patients^6^ recommend age‐specific fasting intervals and supplementation of oral or intravenous glucose solutions during intercurrent illness to prevent catabolism. The dietary intake of LCT should not exceed 25%–30% of the total energy intake and MCT should account for 20% of total fat energy intake.^6^ An age‐specific daily intake of walnut oil is recommended to prevent EFA deficiency. Recent reports suggest less strict dietary treatment for mild phenotypes^7^ and several novel therapeutic substances have been proposed such as Triheptanoin^8^, ^9^ and synthetic ketone esters.^10^

Analysis of acyl carnitines, although useful in detection of the disease, is not as useful for disease monitoring and genotyping is in many cases not indicative of disease severity.^11^ Enzymatic assays determining residual activity of the VLCAD enzyme are more useful in the diagnostic workup. The ETF fluorescence reduction assay^12^ has been used and palmitoyl‐CoA oxidation rate assay utilizing patient lymphocytes^12^ is used in many European countries. The LC‐fatty acid oxidation flux (LC‐FAO flux) method, employed in some countries, measures the oleate oxidation rate in cultured patient fibroblasts.^13^

The incidence as well as the clinical spectrum of VLCADD has changed after the inclusion of this and other fatty acid oxidation disorders in newborn screening programs (NBS) over the last 10–20 years.^14^ An approximately threefold increase in incidence has been associated with a majority of patients remaining asymptomatic during follow up. It is debatable whether the increase in incidence represents detection of mild phenotypes, or children with a benign biochemical phenotype, or is merely a consequence of effective treatment. Since 2010, VLCADD is included in the Swedish NBS program.^15^

Before NBS, only two pediatric patients and three adult patients with VLCADD were known in Sweden but with the introduction of NBS a sharp increase in prevalence was noted. The clinically diagnosed patients displayed severe symptoms and required dietary treatment. The patients detected via NBS displayed few or no clinical symptoms at the time of diagnosis and furthermore the majority showed minor biochemical abnormalities at follow up. The question therefore arose whether Swedish patients identified by NBS are treated with a strict diet including MCT supplementation unnecessarily and if this could even put them in a less favorable position during catabolic situations as suggested by a study in VLCADD (^−^/^−^) mice.^16^

In this study, we present clinical, biochemical, genetic, and screening data from 20 Swedish pediatric patients diagnosed with VLCADD during the first 9 years of NBS and data from two patients diagnosed clinically before the implementation of the expanded screening program. In addition, ex vivo residual enzyme activities using the palmitoyl‐CoA oxidation rate assay were analyzed and correlated to clinical symptoms and newborn screening data.

## 2 MATERIALS AND METHODS

### 2.1 Study population and NBS

Twenty of twenty‐five patients with VLCADD detected by Swedish NBS between November 2010 and November 2019 and two clinically diagnosed patients were included in the study. Three patients detected through NBS declined participation in the study and two patients had moved abroad and were lost to follow‐up. The patients were identified using the Swedish national registry for inborn errors of metabolism (www.rmms.se) and contacted via their physicians.

Clinical data were collected from medical charts and clinical assessment of the patients. Screening was performed at the Swedish National Newborn Screening Laboratory at the Center for Inherited Metabolic Diseases, Karolinska University Hospital, Stockholm, Sweden. Collaborative Laboratory Integrated Reports (CLIR; https://clir.mayo.edu) analysis was performed retrospectively using the results from primary and follow up dried blood spots (DBS). Genetic and biochemical analyses were performed at the Center for Inherited Metabolic Diseases, Stockholm. Lymphocyte residual enzyme activity analysis using the palmitoyl‐CoA oxidation rate‐assay was performed at the University of Freiburg, Germany.

The Swedish newborn screening is performed after 48 h after birth and all DBS are sent to the national screening laboratory. Screening markers used for VLCADD included the levels of C14:1 and the C14:1/C2 ratio, with C14:1 > 0.39 μmol/L and the C14:1/C2 ratio ≥ 0.015 among other metabolites according to R4S/CLIR, reruns in duplicates were performed and evaluations using R4S/CLIR were done to decide whether a child should be recalled for a clinical follow up.^15^, ^17^ VLCADD screening positive cases were reported to one of the four Swedish pediatric metabolic centers for diagnostic tests and clinical evaluation by the attending metabolic physician. True positive cases were confirmed with measurements of plasma acyl carnitines and *ACADVL* gene sequencing and/or analysis of VLCAD residual enzyme activity below the reference interval of healthy controls.

The national screening laboratory joined the collaborative screening database Region 4 Stork project (R4S) in 2010^18^ and subsequently all positive screening cases were analyzed using the R4S database. In 2018, the R4S system was replaced by a second generation of the software, CLIR, inclusive of covariate adjustments such as age at sampling in hours, gestational age, birth weight, and sex.^19^ CLIR consists of a web‐based interactive database of NBS results from multiple screening sites and is used to improve NBS performance.

Plasma acyl carnitines were analyzed using tandem mass spectrometry on either a Xevo TQ or Xevo TQ‐S from Waters, using a modified protocol by Goshal et al.^20^ Measurements of creatine kinase (skeletal muscle CK, and myocardial fractions CK‐MB), aspartate aminotransferase (AST), and alanine aminotransferase (ALT) were performed at the local hospital laboratories.

### 2.2 Clinical evaluation of patients

Medical records for all patients were reviewed for biochemical parameters, longitudinal growth, number of emergency room (ER) visits, ultrasonography results, dietary treatment, presence of gastrostomy, and cognitive assessments. The clinical outcome was summarized using a clinical severity score (CSS), as outlined in Table 3. To evaluate both disease complications and disease burden, the following events were factored in the CSS: neonatal symptoms (defined as events during the first month of life) including lethargy, hypoglycemia, and weight loss >10%, episodes of hypoglycemia after the neonatal period, elevated plasma CK levels, ≥3 episodes of CK‐elevation, elevated transaminases, elevation of both CK and transaminases, rhabdomyolysis (defined as CK more than six times the upper reference level, >36 microkat/L), ≥3 episodes of rhabdomyolysis, admittance to pediatric intensive care unit (PICU), ≥3 ER visits during 1st year of life, strict diet (defined as dietary restriction with LCT <15% of total daily energy intake), placement of gastrostomy tube and night feeds, motor impairment, developmental delay, and epilepsy. Each criterion was given a score of one point resulting in a CSS score ranging from 0 to 15.

### 2.3 Genetic analysis

Genotyping of the *ACADVL* gene (Reference transcript NM_000018.3) was performed at the Center for Inherited Metabolic Diseases. All exons and exon–intron junctions were sequenced from amplified genomic DNA, using the Big Dye Terminator v3.1 Cycle Sequencing Kit (Applied Biosystems) and a DNA analyzer (model 3130 or 3500XL, Applied Biosystems). MLPA analysis was performed using the SALSA MLPA Probemix P076 (MRC‐Holland).

### 2.4 Residual VLCAD enzyme activity analysis

Residual enzyme activity was determined, ex vivo, using purified lymphocytes from 13 out of the 22 patients with the palmitoyl‐CoA oxidation rate‐assay at the University of Freiburg, Germany. Palmitoyl‐CoA oxidation rate was measured as described previously^11^ with minor modifications. The turnover rate of palmitoyl‐CoA was calculated using the sum of the peak area of the products C16:1‐CoA and C16:OH‐CoA and compared with the peak area of the substrate C16:0‐CoA set at an initial concentration of 20 nmol. The enzyme reaction was performed in Tris–HCl buffer (pH 8.0) at 37°C with an incubation time of 5 min. All tests were run in triplicate in the presence of two negative controls chosen randomly from the labs pool of test results. The residual activity of the patients was expressed as percent of the mean value of all healthy controls.

### 2.5 Statistical analysis

Statistical analysis was performed using the IBM SPSS Statistics version 26 (IMB Corporation). Data were presented using descriptive statistics with means and standard deviation. Correlation analysis was performed using the Spearman's rank correlation coefficient test.

## 3 RESULTS

### 3.1 Study population and results from the newborn screening

The 22 study participants (17 male, 5 female) had a mean age of 5.6 years, range 0.3–12.1 years (Table 1). Twelve out of twenty screened patients were diagnosed during the first 2 years after the initiation of the expanded screening. The majority of the NBS samples were taken at the second or third day of life in accordance with the national screening guidelines. During the study period there were, to our knowledge, no false negative cases identified in Sweden.

**TABLE 1:** Patient cohort characteristics

Characteristic	Female	Male	Total
Gender	5 (23%)	17 (77%)	22
Mean age at time of study (years)	4.7	5.9	5.3
Age distribution			
0–3 years	1	4	5
3–6 years	2	1	3
6–9 years	2	10	12
9–12 years	–	2	2

The C14:1 and C14:1/C2 values from the screening samples (NBS1, collected at 48–72 h, mean 58 h) and the results of the NBS samples taken at referral (NBS2, collected at 95–243 h, mean 169 h) are summarized in Table 2. The mean C14:1 level of the follow up samples was significantly lower than for the initial screening samples (C14:1 0.72 ± 0.90 μmol/L versus 3.1 ± 4.4 μmol/L, *p* < 0.001). Seven patients had markedly low C14:1‐levels in the follow up sample and one patient also had very low both C14:1 and C14:1/C2 ratio at the time of referral. The screening results including CLIR scores for both NBS samples are summarized in Table 2.

**TABLE 2:** Genetic mutations, residual enzyme activity, and screening data

Case	Allele 1	Allele 2	Enzyme activity Palmitoyl‐CoA Oxidation rate^a^	C14:1 NBS1	C14:1/C2 NBS1	C14:1 NBS2	C14:1/C2 NBS2	CLIR score NBS 1	CLIR score NBS 2	CSS
**Homozygotes for the common mutation c.848**T>**C**										
1	c.848T>C p.(Val283Ala)	c.848T>C p.(Val283Ala)	–	2.01	0.06	0.42	0.02	572	0	1
2	c.848T>C p.(Val283Ala)	c.848T>C p.(Val283Ala)	–	1.08	0.08	0.23	0.03	313	0	2
3	c.848T>C p.(Val283Ala)	c.848T>C p.(Val283Ala)	19	3.56	0.21	0.74	0.11	1024	588	1
4	c.848T>C p.(Val283Ala)	c.848T>C p.(Val283Ala)	–	2.5	0.12	0.51	0.05	625	212	0
5	c.848T>C p.(Val283Ala)	c.848T>C p.(Val283Ala)	–	0.98	0.08	0.46	0.09	347	214	0
6	c.848T>C p.(Val283Ala)	c.848T>C p.(Val283Ala)	9.9	2.31	0.10	0.21	0.02	816	44	3
7	c.848T>C p.(Val283Ala)	c.848T>C p.(Val283Ala)	–	2.18	0.06	0.35	0.03	761	94	1
**Compound heterozygotes for the common mutation c.848**T>**C**										
8	c.848T>C p.(Val283Ala)	c.335delT p.(Phe113fs)	4.6	6.53	0.2	3.4	0.27	1217	1182	5
9	c.848T>C p.(Val283Ala)	c.896A>T p.(Lys299Met)	–	1.52	0.13	0.19	0.03	650	0	5
10	c.848T>C p.(Val283Ala)	c.896A>T p.(Lys299Met)	–	3.15	0.15	–	–	860	–	2
11	c.848T>C p.(Val283Ala)	**c.963C>A p.(Asn321Lys)** ^**^	–	–	–	–	–	–	–	8
12	c.848T>C p.(Val283Ala)	c.1699C>T p.(Arg567Trp)	–	0.55	0.05	0.75	0.08	135	83	3
13	c.848T>C p.(Val283Ala)	c.1816T>C p.(Trp606Arg)*	4.5	5.96	0.27	0.62	0.06	1188	261	3
14	c.848T>C p.(Val283Ala)	c.1837C>T p.(Arg613Trp)	6.6	8.36	0.33	1.35	0.21	1214	928	4
15	c.848T>C p.(Val283Ala)	c.1837C>T p.(Arg613Trp)	9.4	4.6	0.2	0.9	0.14	1083	615	2
16	c.848T>C p.(Val283Ala)	c.1838G>A p.(Arg613Gln)	12.5	1.2	0.06	0.45	0.04	457	86	3
17	c.848T>C p.(Val283Ala)	**c.1838G**>C p.(Arg613Pro)*	7.4	4.28	0.15	2.34	0.25	1165	1086	5
**Other mutations**										
18	c.343delG p.(Glu115fs)	c.343delG p.(Glu115fs)	1.1	–	–	–	–	–	–	11
19	c.896A > T p.(Lys299Met)	c.1894C>T p.(Arg632Cys)	54.1	1.29	0.05	0.44	0.02	311	0	0
20	c.1238T>C p.(Ile413thr)*	c.1837C>T p.(Arg613Trp)	17.9	2.27	0.08	–	–	589	–	1
21	c.1591C > T; p.(Arg531Trp)	c.1678+1G>C* p.(?)	36.9	1.04	0.04	0.21	0.01	260	0	2
22	c.708‐709delCT p.(Cys237fs)	–	37.1	3.22	0.18	0.18	0.02	940	0	3

*Note*: *Previously not described mutations; **previously not described mutation, p.(Asn321Asp) has been described previously.^21^

^a^ Percentage of healthy controls.

### 3.2 Clinical outcome

No prenatal fetal complications were reported. One patient was born pre‐term at gestational week (GW) 34 and one was born small for gestational age (SGA). Mean birthweight corrected for GA was 0.2 SDS (range − 1.4 to 1.5 SDS). Reported maternal complications were hyperemesis (1), maternal pneumonia (1), mild preeclampsia (1). Few neonatal symptoms were reported such as hypoglycemia (1), lethargy (2), and weight loss exceeding 10% of the birth weight (1).

All 22 patients were examined with echocardiogram (ECG) on at least one occasion, and none displayed cardiomyopathy or episodes of arrhythmia. Elevated CK levels (13/22) and liver transaminases (15/22) were the most common clinical findings after the neonatal period. Three patients had episodes of rhabdomyolysis defined as (CK > 36 microkat/L) (Table 3). One of the clinically diagnosed patients (nr 18) developed frequent rhabdomyolysis episodes from 6 years of age which required intravenous glucose infusion and forced diuresis. The other clinically diagnosed patient (nr 11) had a severe decompensation at 15 months of age precipitated by an infection and developed motor impairment, epilepsy and developmental delay. A total of three patients were diagnosed with attention deficit hyperactivity disorder (ADHD), attention deficit disorder (ADD), autism spectrum disorder, or a combination of these diagnoses. The frequency of ER visits declined with age and most visits were during the first 2 years of life. Four patients had ≥3 ER visits during the first year of life. Details on ER visits are presented in Figure SS1. The mean combined CSS was 3 ± 2.7, range 0–11. Three patients had a score of 0 and were considered asymptomatic, two patients had a score ≥8 and were considered severely affected (Table 3).

**TABLE 3:** Clinical severity score

	Clinical Symptoms			Hospitalization			Biochemical findings			Treatment		Clinical severity Score
Case	Motor impairment/Developmental delay/Epilepsy	Neonatal symptoms^a^	ER visits 1st year of life (no)	PICU admittance (no)	Hypoglycemia (no)^b^	Elevated CK (no)^c^	Rhabdomyolysis (no)^d^	Elevated transaminases (no)^e^	Elevated transaminases and CK (no)	Gastrostomy and Nightfeeds	Strict diet^f^	CSS (0–15 points)^g^
1	–		–	–	–	1	–	–	–	–	–	**1**
2	–		2	–	–	1	–	4	–	–	–	**2**
3	–		1	–	–	–	–	3	–	–	–	**1**
4	–		2	–	–	–	–	–	–	–	–	**0**
5	–		1	–	–	–	–	–	–	–	–	**0**
6	–		2	–	–	1	–	6	–	–	1	**3**
7	–		1	–	–	–	–	–	–	–	1	**1**
8	–		2	–	–	5	1	5	–	–	1	**5**
9	–		3	–	–	1	1	1	–	–	1	**5**
10	–		1	–	–	1	–	5	–	–	–	**2**
11	MI, DD, E		1	1	1	2	–	4	–	1	–	**8**
12	–		2	–	–	2	–	1	1	–	–	**3**
13	–	L	1	–	–	–	–	2	–	–	1	**3**
14	–	L	4	–	–	–	–	1	–	1	1	**4**
15	–		1	–	–	2	–	–	–	–	1	**2**
16	–		2	–	–	1	–	1	1	–	–	**3**
17	–		10	–	1	2	–	–	–	1	1	**5**
18	–	H	2	1	1	39	17	46	35	1	1	**11**
19	–		–	–	–	–	–	–	–	–	–	**0**
20	–		1	–	–	–	–	1	–	–	–	**1**
21	–		1	–	–	1	–	3	–	–	–	**2**
22	–	W	3	–	–	–	–	3	–	–	–	**3**
												

^a^ Neonatal symptoms registered in patient medical chart: lethargy (L), weight loss >10% of birth weight (W), hypoglycemia <2.6 mmol/L (H).

^b^ Hypoglycemia: recorded episode with blood glucose <2.5 mmoL/L.

^c^ Elevated creatinine kinase (CK): recorded episode with CK > 6 microkat/L.

^d^ Rhabdomyolysis: recorded episode with CK > 36 microkat/L.

^e^ Elevated transaminases (AST and ALT): recorded episode with AST > 0.75 microkat/ L and/or ALT>1.2 microkat/L.

^f^ Strict diet: dietary restriction with LCT <15% of total daily energy intake.

^g^ Clinical severity score (CSS): 0–15 points. 1 point each for: Hypoglycemia, 1 episode of elevated CK, ≥3 episodes of CK‐elevation, 1 episode of elevated transaminases, 1 episode of both elevated CK and transaminases, 1 episode of rhabdomyolysis, ≥3 episodes of rhabdomyolysis, 1 admittance to PICU, ≥3 ER visits during 1st year of life, strict diet (LCT < 15%), gastrostomy + night feeds, presence of motor impairment, developmental delay, epilepsy, neonatal symptoms.

Nineteen out of twenty‐two patients were maintained on a fat‐restricted diet and 18 patients were prescribed MCT supplementation. In the treated group, fat restriction measured as percentage of LCT fat intake in relation to total daily energy intake (TDI) ranged from 4% to 27% with a mean value of 20.4 ± 5.4%. Nine patients had less than 15% of LCT intake of the TDI and which was considered a strict diet. Four patients had a gastrostomy and were given night feeds. All patients were prescribed a dextrinomaltose supplement (Fantomalt®) and were provided with a written emergency regimen instruction.

**FIGURE 1:**
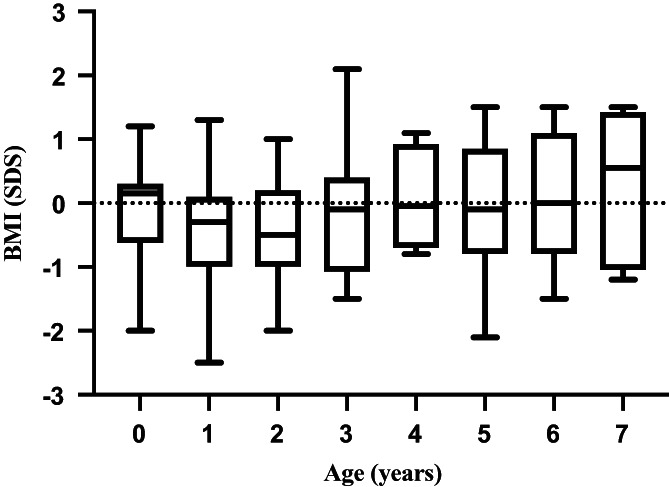
Cohort BMI standard deviation score

All patients were within +/−2.5 *SD* for height and weight except one patient. The mean ISO‐BMI for the cohort is summarized in Figure 1. ISO‐BMI was slightly below Swedish population mean values at the age of 1 and 2 years but did not differ from Swedish population mean values between the ages of 3–7 years (Figure 1).

Genotyping showed the c.848T>C (p.Val283Ala) mutation of the *ACADVL* gene to be the most prevalent disease causative variant. Eight patients were homozygous and another eight were compound heterozygous for this mutation (Table 2). Five patients carried novel mutations and four of those were compound heterozygous for the c.848T>C (p.Val283Ala). All novel mutations were predicted to be pathogenic by in silico analysis. One patient was heterozygous for the c.708_709delCT mutation (nr 22). MLPA‐analysis in this patient did not detect any deletions or duplications.

Residual VLCAD enzyme activity analysis was performed in lymphocytes obtained from the subgroup of 13 patients using the palmitoyl‐CoA oxidation rate‐assay. Enzyme activity ranged between 1.1% and 54.1%. Mean enzyme activity was 17% (range 1.1%–54.1%) (16) of wild‐type enzyme activity (Table 2).

### 3.3 Correlation between the CLIR score, the CSS, and residual enzyme activities

**FIGURE 2:**
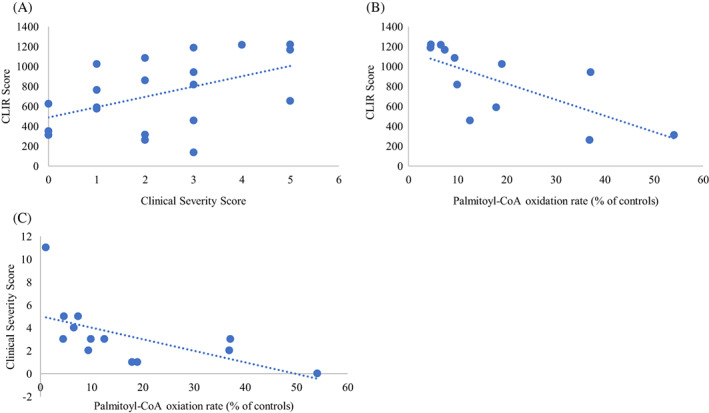
Correlation between CSS, CLIR score, and residual enzyme activity in patient lymphocytes. (A) Spearman’s rho correlation coefficient 0.486, *p* = 0.043. (B) Spearman’s rho correlation coefficient −0.830, *p* = 0.016. (C) Spearman’s rho correlation coefficient −0.769, *p* = 0.003

Spearman rank‐order correlation coefficient test showed a positive correlation between CLIR score and the CSS (0.486, *p* = 0.043) and a negative correlation to the Palmitoyl‐CoA oxidation rate (−0.830, *p* = 0.016). A negative correlation between the CSS and residual enzyme activity measured by the palmitoyl‐CoA oxidation rate was found (−0.769, *p* = 0.003) (Figure 2).

## 4 DISCUSSION

This study presents a national cohort of patients with VLCADD diagnosed in Sweden during 2010–2019 via NBS or due to clinical symptoms prior to the introduction of screening for VLCADD. As a group, the patients identified via NBS showed no or mild symptoms and had normal growth. The clinically diagnosed patients born before NBS had a more severe phenotype and were diagnosed after decompensation or due to neonatal symptoms.

Introduction of NBS and diagnosis of presymptomatic patients has caused a clinical dilemma regarding which of these patients require a strict dietary treatment. This study indicates that taken together, the CLIR score, the residual enzyme activity, and genetic variants may provide guidance for early clinical decisions.

Four out of twenty‐two patients had neonatal symptoms. Maternal HELLP syndrome, which is associated with pregnancies with infants affected with long chain 3‐hydroxyacyl‐CoA dehydrogenase deficiency (LCHADD)^22^ was not reported in this study. Three patients (nr 11,17,18) had at least one episode of hypoglycemia but only one episode was recorded during the neonatal period. These findings are comparable to other reported NBS cohorts.^23^, ^24^ The low frequency of hypoglycemia was not surprising since this is usually a late sign of metabolic decompensation in VLCADD.^25^ Only three patients (nr 8,9,18) had episodes of rhabdomyolysis defined as CK‐levels >36 microkat/L. Previous reports on increased frequency of muscle symptoms and rhabdomyolysis in older children with VLCAD deficiency^26^ suggest that the young patients in this cohort might be at risk of more frequent rhabdomyolysis later in life. Indeed, one of the older clinically diagnosed patients (nr 18) had frequent episodes of rhabdomyolysis starting from 6 years of age despite having mainly mild symptoms prior to that age.

Most patients had more frequent ER visits during the first years of life and the frequency of visits declined with age (Figure SS1). This is likely due to the increased risk of contracting viral infections in younger children, the inability to sustain adequate energy intake via oral emergency regimen, and parental worry rather than age‐correlated disease specific factors.

The clinical outcome of NBS detected patients with VLCADD in Spain,^27^ Utah,^24^ and the Netherlands^23^ are similar to the Swedish experience in many ways. The common findings are a high proportion of cases with compound heterozygosity for mutations in the *ACADVL* gene, benign clinical features and few cases with an early onset phenotype.

The majority of the patients in the study had dietary treatment although the percentage of LCT fat in relation to the TDI varied from a very strict fat reduction resembling that of LCHADD treatment to more relaxed regimens. Concerns have been raised that the high caloric, carbohydrate rich diet prescribed in several beta oxidation disorders might lead to obesity, and an increased risk of insulin resistance and liver lipid deposition. A Swedish study on pediatric patients with LCHADD showed increased BMI‐SDS compared to the general population.^28^ In the present VLCADD study mean ISO‐BMI was slightly lower compared to the Swedish population mean at 1 and 2 years of age and from age 3 to 7 years it was comparable to the Swedish population mean (Figure 1). The fact that all Swedish patients with LCHADD are prescribed night feeds versus only three out of our patients with VLCADD may contribute to the difference in BMI‐SDS outcome. There were no reports of type 2 diabetes or pre‐diabetes in the cohort but fasting insulin and fasting glucose measurements were not part of the standard clinical work up. The short follow‐up time and low mean age in this cohort warrants further studies regarding cardiovascular risk factors later in life.

The molecular genetic results in our patients are similar to what has previously reported in North American cohorts.^24^, ^29^ The most common mutation, c.848T>C, was present in >50% of all analyzed alleles (Table 2). The patients homozygous for c.848T>C had CSS from 0 to max 3 reflecting a relatively benign clinical course. Most cases compound heterozygous for c.848T>C and another variant show benign clinical features but a few cases with an early onset phenotype have been described in Spain,^27^ Utah,^24^ and the Netherlands.^23^ Thus, for these cases, the genetic findings confirm the VLCADD diagnosis but cannot alone support decisions for the clinical management. Patients with null mutations had a more severe clinical phenotype. The patient homozygous for the null mutation c.343delG (nr 18) had frequent decompensations with rhabdomyolysis and the patient compound heterozygous for c.848T>C and the null mutation c.335delT (nr 8) had several episodes with increased levels of CK during infections. Both these patients had residual enzyme activities below 5% in the palmitoyl CoA oxidation rate analysis. The patient with only one identified mutation (nr 22) had a mild clinical picture and an enzyme activity (palmitoyl CoA flux activity) of 37%.

The importance of the timing of NBS testing^30^ and the usefulness of including the C14:1/C2 ratio and other markers and ratios from R4S and CLIR was demonstrated in our report. Seven out of 20 NBS patients had markedly lower C14:1‐ levels in the follow‐up sample and only one patient had both C14:1 and C14:1/C2 levels above rerun (Table 2). CLIR scores showed a similar pattern with declining scores between the screening and follow up sample (Table 2). The skewed sex ratio, 17 out of 22 cases were males in our cohort, likely represents statistical chance due to the low number of participants, as a recessive disorder should affect both sexes equally. Earlier reports have shown female ratios to be higher^24^ or more equal.^29^ The prevalence of VLCADD in Sweden is estimated to be 1:42.000^15^ which is in line with several other publications.^24^, ^30^

Surprisingly, 12 of 20 NBS patients in our cohort were diagnosed during the first 2 years of screening. The R4S and later the CLIR post analytical interpretive tools have been used, but only eight patients were diagnosed during the following 6 years. However, we could not identify any difference in residual enzyme activity or the CSS between the early and later screening detected patients. Hence, the spectrum of disease among the first 12 patients did not seem to differ from the later detected patients.

One of the main concerns of VLCADD screening has been the difficulty to predict clinical outcome based on genetic and residual enzyme activity data which in many cases are available already in the newborn period. Diekman et al.^31^ showed a correlation between LC‐FAO flux data and a combined CSS based on the prevalence of hypoglycemia, cardiomyopathy and myopathy in a Dutch cohort of patients with VLCADD. In the present study, additional factors such as neonatal symptoms, frequent ER admissions, and night feeds were included in the CSS in order to reflect disease burden. Our results show a moderate to strong correlation between the CSS and the enzyme activity (Figure 2). Interestingly, the CLIR scores from the screening samples showed a strong correlation to the palmitoyl‐CoA‐oxidation rate but only a moderate correlation to the CSS (Figure 2). This may at least in part be due to the fact that the two severely affected clinically diagnosed patients could not be included in this analysis since they had not been screened. The low number of patients with this rare disease and the fact that only two clinically diagnosed patients were included in the study makes comparisons between groups challenging. The enzyme activity measurements were available in a subgroup of the patients, 13 out of 22 patients. The close collaboration between Swedish pediatric metabolic centers and a nation‐wide electronic medical chart system, however, allowed for a very detailed record of clinical data to be compiled.

The identified correlations between the CLIR scores, residual enzyme activity, and the CSS suggest that information already available in the newborn period might be useful in predicting disease severity. This observation however needs to be confirmed in a larger study. As patients grow older, assessment of the cognitive outcome over time will be an essential part of the evaluation.^32^ In addition, further studies on the effect of the dietary treatment per se on metabolism and cardiovascular risk factors in patients with VLCADD are needed.

## 5 CONCLUSION

This national cohort study shows a wide range of clinical outcomes in Swedish patients with VLCADD varying from asymptomatic to severely affected. There is a clear difference between the clinically diagnosed patients who had severe symptoms early in life and the NBS group, although heterogenous, which displayed milder courses of disease. The screening CLIR score in combination with the residual enzyme activity, and genetic analysis may be useful in predicting disease severity and the need for dietary treatment already in the neonatal period. Larger follow‐up studies to verify our findings are warranted.

## CONFLICT OF INTERESTS

The authors declare that they have no conflict of interests.

## AUTHOR CONTRIBUTIONS

David Olsson, Anna Nordenström, Maria Halldin, Svetlana Lajic, Sara Tucci, Rolf H. Zetterström, Michela Barbaro, and Charlotte Haglind wrote the manuscript. David Olsson, Anna Nordenström, Maria Halldin, and Svetlana Lajic designed the research. Sara Tucci performed residual enzyme analysis. Michela Barbaro performed the genetic analysis. David Olsson performed the data collection. David Olsson, Anna Nordenström, Maria Halldin, Svetlana Lajic, Rolf H. Zetterström, and Michela Barbaro analyzed the data.

## ETHICS STATEMENT

This multi‐center study was approved by the ethical board of Uppsala University registration number 2006:005. All procedures followed were in accordance with the ethical standards of the responsible committee on human experimentation (institutional and national) and with the Helsinki Declaration of 1975, as revised in 2000. Informed consent was obtained from all patients for being included in the study.

## Supporting information


**Appendix Figure S1** Frequency of ER‐visits

## Data Availability

All original data supporting the reported results can be obtained by contacting the corresponding author.
