# Supplementary figures and images for: Very long‐chain acyl‐CoA dehydrogenase deficiency in a Swedish cohort: Clinical symptoms, newborn screening, enzyme activity, and genetics
Source: JIMD Rep. 2022 Jan 9;63(2):181–90. doi: 10.1002/jmd2.12268 (PMC8898720; doi:10.1002/jmd2.12268)

Appendix 1: Frequency of ER visits per year

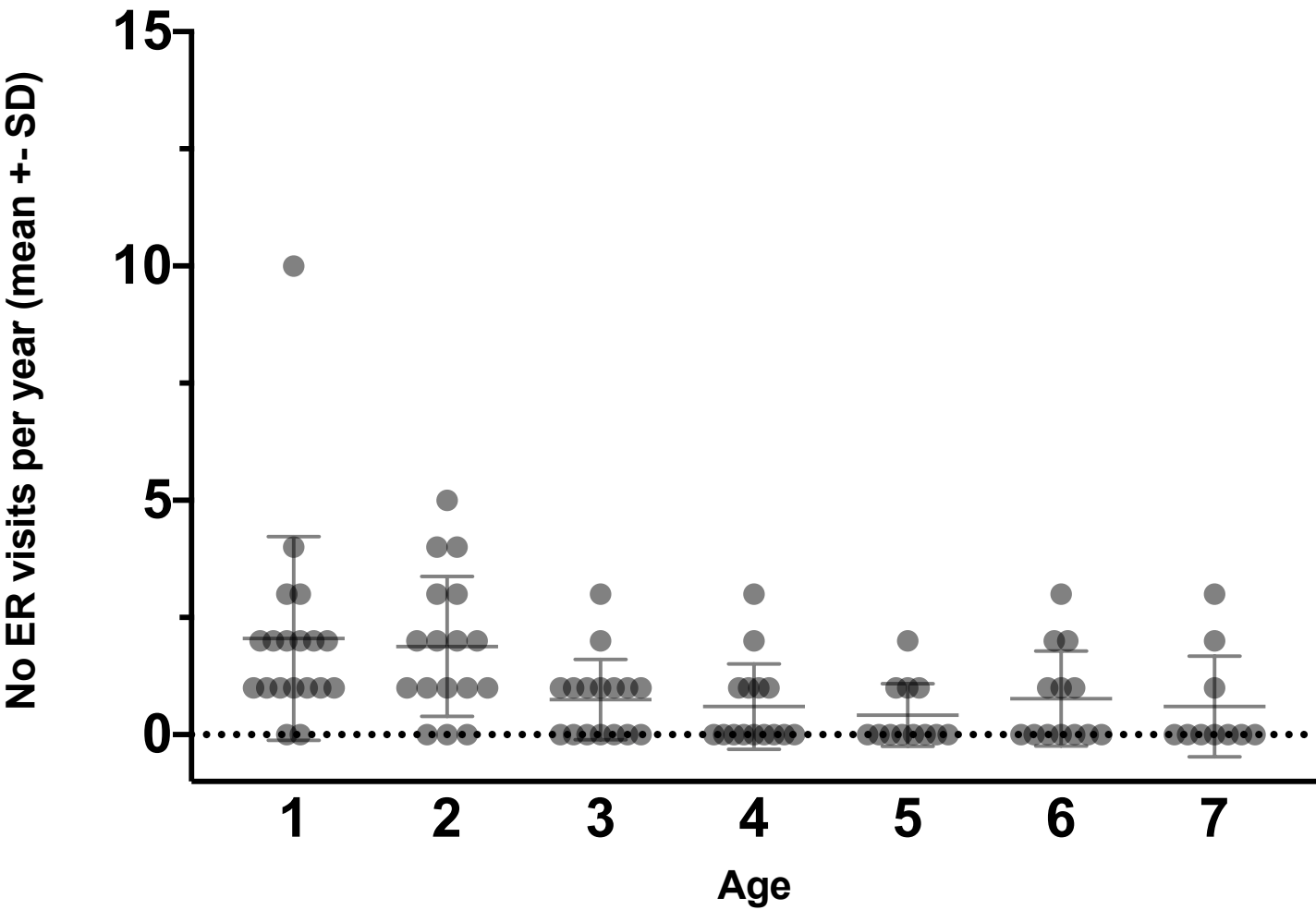

Supplement: Supplementary file 1 — Appendix Figure S1 Frequency of ER‐visits [file JMD2-63-181-s001.pdf]
